# Retraction: Association Between Ureteral Double J Stent Colonization and Lower Urinary Tract Symptom Severity: A Cross-Sectional Study

**DOI:** 10.7759/cureus.r46

**Published:** 2022-03-17

**Authors:** Malik Hatim Hussain, Daniyal Jilanee, Mustafa Naeem, Syed Usama Ashraf, Camilo A Avendaño-Capriles, Shivani Mehta, Syed Ali H Zaidi, Syed Kumail H Zaidi, Sohaib Tousif, Rahil Barkat

**Affiliations:** 1 Orthopaedics and Trauma, East Lancashire NHS Hospitals, Blackburn, GBR; 2 Medicine, Liaquat National Hospital and Medical College, Karachi, PAK; 3 Medicine, Dow University of Health Sciences, Karachi, PAK; 4 Foundations of Clinical Research (FCR) Program Scholar, Harvard Medical School, Boston, USA; 5 Medicine, Universidad del Norte, Barranquilla, COL; 6 Medicine, Xavier University School of Medicine, Oranjestad, ABW; 7 Medicine, Jinnah Medical and Dental College, Karachi, PAK; 8 Medicine, Dow International Medical College, Karachi, PAK; 9 Medicine, Ziauddin Medical University, Karachi, PAK; 10 Indus Hospital Research Center, The Indus Hospital, Karachi, PAK

This article has been retracted due to the unknown origin of the data, lack of verified IRB approval, and purchased authorships. The primary author, Rahil Barkat, was involved in data theft and misuse in two recently published Cureus articles, which have since been retracted.

As the origin of this article’s data and verified IRB approval cannot be confirmed, we have made the decision to retract this article. Cureus has confirmed that the co-authors were asked by Mr. Barkat to proofread the article and provide payment in exchange for authorship. (Proofreading is an insufficient contribution to warrant authorship as defined by ICMJE.) These payments were made in the guise of “editing fees” but greatly exceed any editing fees paid to Cureus. While these authors may have been defrauded by Mr. Barkat, they remain complicit due to their lack of honest contributions to the article.

